# Dynamic Metabolic Changes during the First 3 Months after ^90^Y-Ibritumomab Tiuxetan Radioimmunotherapy

**DOI:** 10.1155/2014/368947

**Published:** 2014-06-19

**Authors:** Miwako Takahashi, Toshimitsu Momose, Keitaro Koyama, Motoshi Ichikawa, Mineo Kurokawa, Kuni Ohtomo

**Affiliations:** ^1^Department of Radiology, Graduate School of Medicine, The University of Tokyo, 3-1 Hongo 7-Chome, Bunkyo-ku, Tokyo 113-8655, Japan; ^2^Department of Hematology and Oncology, Graduate School of Medicine, The University of Tokyo, 3-1 Hongo 7-Chome, Bunkyo-ku, Tokyo 113-8655, Japan

## Abstract

*Objective*. To elucidate the time course of tumor metabolism during the first 3 months after ^90^Y-ibritumomab tiuxetan radioimmunotherapy (RIT) in patients with refractory malignant lymphoma. *Materials and Methods*. Seven patients with recurrent follicular lymphoma underwent FDG-PET imaging before and after 1-, 4-, and 12-week RIT with ^90^Y-ibritumomab tiuxetan. Tumor metabolic activity on FDG-PET scans was assessed as the maximum standard uptake value (SUVmax). *Results*. Decrease in metabolism was detected 1 week after RIT. In the most decreased lesion, SUVmax decreased to 20% of the baseline value during the first week. Most lesions continued to decrease for up to 4 weeks. Some lesions showed increased metabolism from 4 to 12 weeks, while the level of FDG accumulations at 12 weeks was still lower than the baseline. *Conclusions*. Tumor response to RIT could be observed as early as 1 week after the administration of RIT. After tumor activity decreases, the metabolism may increase at least between 4 and 12 weeks. It suggests that the metabolic changes should be carefully evaluated during this period.

## 1. Introduction

With advances of various antitumor drugs, such as chemotherapy, molecular-targeting drug, and radioimmunotherapy (RIT), treatment option is increasing for patients with malignant lymphoma. In this kind of situation, accurate monitoring of tumor responses is important to differentiate between patients who are responders to the treatment from nonresponders who will need further therapy. For evaluating tumor response, measurements of different aspects of tumor, such as tumor marker, immunopathological studies, and radiological imaging examinations, have been applied [1]. Among them, ^18^F-fluorodeoxyglucose- (FDG-) positron emission tomography (PET) more directly and less invasively reflects tumor activity by visualizing tumor glucose metabolism and is now widely conducted in evaluation of treatment effect [2, 3]. However, as compared to other modalities, frequency of performing FDG-PET is limited because it is relatively expensive and patient is exposed to injected FDG. Therefore, we need to determine the timing of FDG-PET scans for monitoring tumor responses. Currently, the idea that the evaluation of treatment effect should be performed in the early period after chemotherapy has been focused on because metabolic changes in tumor are documented by FDG-PET and its responses are relatively well correlated with long-term outcome [4–9]. Therefore, the timing should be considered including the early period after the initiation of therapy.

^90^Y-Ibritumomab tiuxetan, one of the RITs, has emerged in clinical practice as a treatment for patients with refractory malignant lymphoma. ^90^Y-Ibritumomab tiuxetan is the pure *β*-emitter ^90^Y conjugated to a murine IgG monoclonal antibody targeting the CD20 antigen [10]. ^90^Y-Ibritumomab tiuxetan has been shown to produce favorable outcomes in patients who were refractory to chemotherapy including rituximab [11–13]. Several investigations of FDG-PET study on tumor response to ^90^Y-ibritumomab tiuxetan have been reported, but the time course until 2 months after the administration has not been reported. The characteristics of time course of metabolic changes, such as how early the metabolic changes occur after the drug administration or when the tumor activity turns to increase after decreases, are helpful to decide the timing of monitoring tumor responses.

In this study, to demonstrate the time course of tumor metabolic changes during the first 3 months after ^90^Y-ibritumomab tiuxetan treatment, we obtained FDG-PET scans before and 1, 4, and 12 weeks after the treatment of patients with refractory follicular lymphoma.

## 2. Materials and Methods

### 2.1. Patients

We evaluated 7 patients with recurrent follicular lymphoma treated with ^90^Y-ibritumomab tiuxetan (Zevalin; FUJIFILM RI Pharma Co., Ltd., Kyobashi, Tokyo) who underwent FDG-PET imaging before and after treatment. The average patient age at the time of treatment was 65.7 years (range, 56–78 years). All 7 patients had received at least 2 prior chemotherapy regimens including rituximab (average, 3.7; range, 2–6), with baseline FDG-PET scans performed in all 7 patients at least 28 days after the completion of the previous chemotherapy regimen. Sixteen posttreatment FDG-PET scans were also examined, including 1-week scans for 4 patients (numbers 1, 2, 3, and 7), 4-week scans for 6 patients (numbers 2–7), and 12-week scans for 6 patients (numbers 2–7). Detailed characteristics of patients are shown in Table 1.

The design of this retrospective study was approved by the Ethics Review Board at our hospital, and all patients provided informed consent.

### 2.2. FDG-PET Protocol

Patients fasted for at least 5 h before undergoing FDG-PET, and a blood sugar level under 150 mg/dL was required. Each patient received 296 MBq of intravenous FDG. Imaging was then performed 50 min later using an Aquiduo PET/CT scanner (Toshiba Medical Systems, Otawara, Japan). This scanner contains 24,336 lutetium oxyorthosilicate (LSO) crystals in 39 detector rings and has an axial field of view of 16.2 cm and 82 transverse slices of 2.0 mm thickness. The intrinsic full width half-maximum (FWHM) spatial resolution in the center of the field of view is ~4.3 mm, and the FWHM axial resolution is 4.7 mm. The sinogram was acquired in the 3-dimensional mode. The CT scan was performed with a tube current of 50 mA and a tube voltage of 120 kV for attenuation correction, and a 2.5 min emission scan per position was acquired. Images were reconstructed using Fourier rebinning ordered subset expectation maximization (OSEM) iterative reconstruction, with 2 iterations and 8 subsets, and a 4 mm FWHM Gaussian filter was applied. The data were collected in a 128 × 128 × 41 matrix with a voxel size of 2.0 × 2.0 × 4.0 mm.

### 2.3. Analysis of Lesion Activity

A circular region of interest (ROI), 1 cm in diameter, was placed on the most intensive FDG uptake area of each lesion. Only lesions ≥1 cm in diameter were evaluated. The maximum standard uptake value (SUVmax) within the ROI (SUV_lesion_max⁡) was used to represent the activity of the lesion.

We also calculated the mean SUV of the blood pool (SUV_BP_mean) by placing a similar ROI on the mediastinal blood pool structure (the ascending aorta). An SUV_lesion_max⁡ greater than the SUV_BP_mean was indicative of PET-positive lesion activity, and lesions for which the SUV_lesion_max⁡ was greater than the SUV_BP_mean were evaluated in this study. The average degree of changes in metabolic activity was calculated relative to baseline value using the following formula:

(1) SUVmax⁡lesionbaseline  SUVmax⁡lesion×100[%].

### 2.4. Radioimmunotherapy Protocol

We used commercially available ^90^Y-ibritumomab tiuxetan kits (Zevalin). Each preparation kit consisted of 3 vials containing ibritumomab tiuxetan, sodium acetate, and buffer solution, as well as an empty reaction vial. ^90^Y-ibritumomab tiuxetan was prepared according to the manufacturer's instructions. First, the inner wall of the reaction vial was coated with sodium acetate; ^90^Y (1500 MBq) and ibritumomab tiuxetan (3.2 mg) were then mixed in the reaction vial and left at room temperature for 5 min to allow for chemical reaction. The reaction was then stopped by addition of buffer solution. The ^90^Y-ibritumomab binding fraction was confirmed to be higher than 95% with thin-layer chromatography. The unlabeled anti-CD20 antibody rituximab (250 mg/m^2^) was administered to improve the targeting of the radio-conjugated antibody by reducing the binding sites of circulating B-cells. ^90^Y-Ibritumomab tiuxetan was then administered at a dose of 14.8 MBq/kg over 10 min. All patients underwent ^111^In-ibritumomab tiuxetan (130 MBq) imaging to confirm the expected biodistribution.

## 3. Results

**Figure 1:**
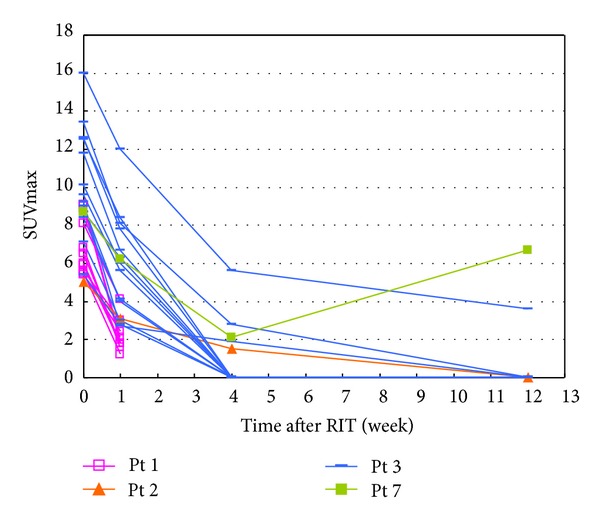
Changes in SUV_lesion_max⁡ as a function of time after RIT in patients 1–3 and 7.

**Figure 2:**
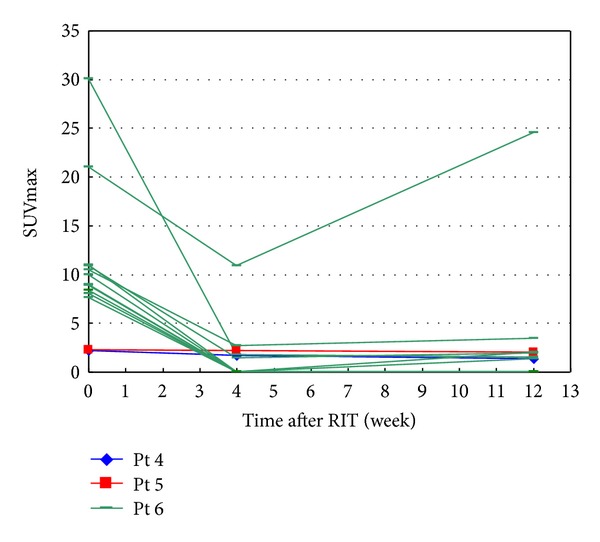
Changes in SUV_lesion_max⁡ as a function of time after RIT in patients 4–6.

**Figure 3:**
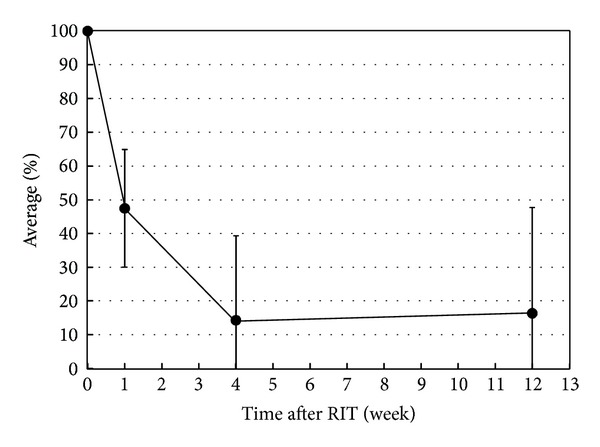
Average percent decrease in SUV_lesion_max⁡ relative to baseline values as a function of time after RIT. Error bars represent standard deviations, with negative error bars on the *y*-axis omitted.

We found that the 7 patients had a total of 38 PET-positive lesions according to baseline FDG-PET scans; of these, 25 lesions, in patients 1, 2, 3, and 7, were evaluable at 1 week after ^90^Y-ibritumomab tiuxetan treatment. The activity of these 25 lesions as a function of time is shown in Figure 1. Activity for all 25 lesions sharply decreased at 1 week and, except for 1 lesion in patient 7, continued to decrease until 12 weeks.  Figure 2 shows the time course of the other 13 lesions, in patients 4, 5, and 6. The activity of all of these lesions was lower at 4 weeks than at baseline and, except for 5 lesions in patient 6, continued to decrease until 12 weeks. The average (standard deviation) degrees of changes in lesion activity at weeks 1, 4, and 12, determined from 25, 28, and 28 lesions, respectively, were 47.5% (17%), 14.3% (25%), and 16.4% (31%), respectively (Figure 3).

**Figure 4:**
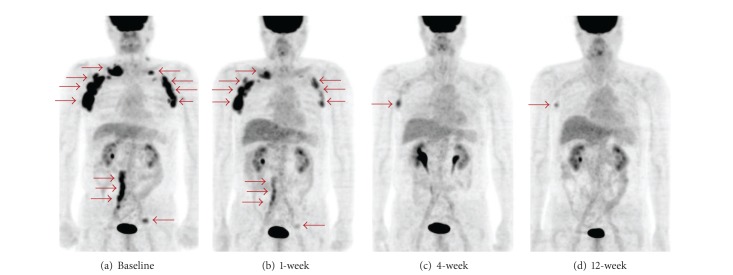
Maximum intensity projection images of patient 3. (a) Baseline, (b) 1-week scan, (c) 4-week scan, and (d) 12-week scan. FDG-avid lesions (arrow) are seen in bilateral subclavicular, axillary, abdominal para-aortic, and left inguinal area at baseline. Decrease in metabolism is observed at 1 week. All lesions continued to decrease to 12 weeks. At 12 weeks, only slight accumulation in the right axillary lesion is visible. Physiological activities are seen in the liver and the urinary tract.

Figure 4 shows FDG-PET images of a representative case (patient 3) with a decreased metabolism from baseline to 12 weeks. Multiple FDG-avid lesions were found in bilateral subclavicular, axillary, abdominal para-aortic, and left inguinal area at the baseline scan. Decrease in metabolism was detected at 1 week. FDG uptakes of all lesions continued to decrease to 12 weeks.

**Figure 5:**
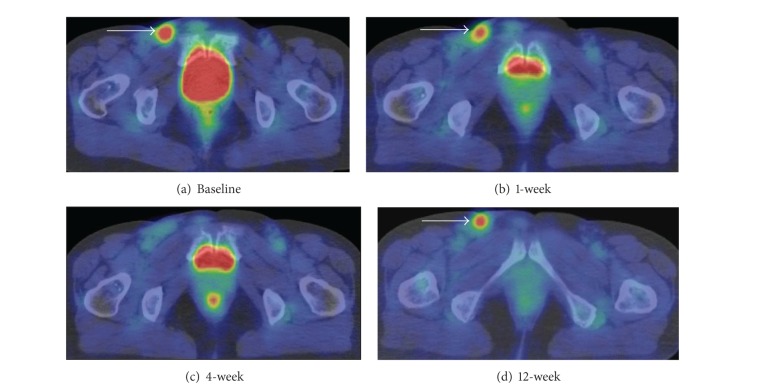
Axial images at level of inguinal region of patient 7. (a) Baseline, (b) 1-week scan, (c) 4-week scan, and (d) 12-week scan. FDG-avid lesion is seen in the right inguinal lesion (arrow). FDG uptake decrease for up to 4 weeks and the degree of the uptake is not remarkable at 4 weeks, but the declining metabolism turns to increase from 4 weeks to 12 weeks. Physiological activities are seen in the bladder and the rectum.

Figure 5 shows FDG-PET images of a case (patient 7) with an increased metabolism from 4 weeks to 12 weeks. The FDG uptake of the right inguinal lesion decreased from the baseline to 4 weeks and turned to increase from 4 weeks to 12 weeks, but the FDG uptake of lesion was still lower than the baseline. This lesion was proven as recurrence histologically after this scan.

## 4. Discussion

We demonstrated the metabolic changes from 7 patients during the first 3 months after RIT with ^90^Y-ibritumomab tiuxetan. Remarkable decrease was observed on FDG-PET at 1 week after the therapy. Most lesions continued to decrease in metabolism for up to 12 weeks, but some lesions turn to increase between 4 and 12 weeks. This result suggests that the tumor metabolism has begun to change within the first week after RIT, and the turning from the first decline to increase in metabolism can occur at least between 4 and 12 weeks.

FDG-PET study on evaluation of tumor response to ^90^Y-ibritumomab tiuxetan has previously reported that the response evaluation obtained from FDG-PET at 8 weeks was corresponding to the findings of those at 24 weeks [14]. Our result that the tendency of tumor activity began to vary between 4 and 12 weeks after RIT seems to explain the consequence of the previous study. Treatment effect depending on lesion may begin to be visible on FDG-PET during this period. One patient (patient 7), shown in Figure 5, has been proven as recurrence at 12 weeks. The degree of uptake was still lower than the baseline, but the tendency of metabolic change was increasing from 4 weeks, which leads to invasive pathological examination. Careful monitoring during this period may contribute to identifying early sign of recurrence among RIT patients.

Early evaluation for patients treated with chemotherapy at 1 cycle or 2 cycles after regimens has been reported to be predictive for long outcome [4–9]. In addition, remarkable decreases in metabolism at 7 days after the initiation of chemotherapy were reported and also related to long-term outcome [15]. Our data, the remarkable decreases at 7 days after RIT, may also provide feasibility of early evaluation in patient with RIT.

On the other hand, early evaluation needs a caution of transient increase in metabolism caused by treatment-related factors [16]. In patients treated with external radiation therapy, FDG-PET is generally used to assess response at 6 to 8 weeks after the completion of the therapy because radiation-induced inflammatory uptake may confound the interpretation of FDG-PET scans [3]. Compared to external radiation therapy, RIT involves the use of lower doses of radiation rather than external radiation therapy. In addition, ^90^Y is a pure *β*-emitter that affects only 100 to 200 cells [17, 18]. Therefore, surrounding normal tissue response is less likely to confound measurement on FDG-PET after RIT. In previous study, FDG uptake transiently increased more than the baseline at 7 days after RIT in one patient who reached complete response with ^131^I-anti-B1 radioimmunotherapy [19]. In our study, no patients showed increases more than the baseline. The clinical interpretation of the transient increases in the early period after treatment remains to be fully elucidated.

The limitations of this study are the small number of patients and that the FDG-PET scan at each time could not be obtained from all patients, but this study is the first to demonstrate the time course of tumor metabolic changes early after RIT, which provides the basis of deciding the timing of FDG-PET scans. In future study, we should compare the tumor response obtained from the FDG-PET monitoring with long outcome in a large number of patients.

## 5. Conclusion

We demonstrated metabolic changes in recurrent follicular lymphomas during the first 3 months after ^90^Y-ibritumomab tiuxetan treatment. Tumor response to RIT could be observed with FDG-PET as early as 1 week after the therapy. Tendency of the metabolic changes begins to vary from 4 to 12 weeks, which suggests that careful monitoring is needed during this period.

## Figures and Tables

**Table 1:** Patient population characteristic.

Pt	Age at onset (years)	Stage at onset	Number of prior regimens	Age at RIT (years)	Stage at RIT	Days after previous treatment (days)	PET scan			
							Baseline	1-week	4-week	12-week
1	60	II	6	76	III	27	◯	◯	n/a	n/a
2	55	III	4	63	I	31	◯	◯	◯	◯
3	48	II	3	61	III	478	◯	◯	◯	◯
4	56	III	2	59	I	68	◯	n/a	◯	◯
5	70	III	3	78	I	87	◯	n/a	◯	◯
6	57	I	4	67	III	90	◯	n/a	◯	◯
7	43	III	4	56	I	143	◯	◯	◯	◯

n/a: not available.
